# Supplementary material for: Selective Survival of Protective Cultures during High-Pressure Processing by Leveraging Freeze-Drying and Encapsulation
Source: Foods. 2022 Aug 16;11(16):2465. doi: 10.3390/foods11162465 (PMC9407356; doi:10.3390/foods11162465)
Supplement: Supplementary file 1 [file foods-11-02465-s001.zip › foods-1832813-supplementary.pdf]

Selective survival of protective cultures during high-pressure processing by leveraging freeze-drying and encapsulation.

Meghan R. McGillin*, Dana L. deRiancho, Timothy A. DeMarsh, Ella D. Hsu, Samuel D. Alcaine*

Department of Food Science, Cornell University, Ithaca NY 14850
* Correspondence: mrm392@cornell.edu; M.M.; alcaine@cornell.edu; S.A.

SKIM MILK SAMPLE	SPECIES	ACCESSION NO.
IC-SM/4	*Bacillus thuringiensis*	CP050183
IC-SM/24	*Bacillus thuringiensis*	CP050183
IC-SM/72	*Bacillus subtilis*	KR967391
EN-SM/24	*Bacillus subtilis*	MN512155
EN-SM/72	*Bacillus cereus*	OK655836

SI Table S1. Environmental contamination PCR identification. Preliminary fermentation trials had utilized pasteurized skim milk as the immersion medium repeatedly showed high levels of contamination by *Bacillus* spore formers generally known to be native to this substrate. Species identification was achieved by polymerase chain reaction amplification of their 16S ribosomal RNA genes, followed by Sanger sequencing; the resulting sequences were then compared to those in the National Center for Biotechnology Information's Nucleotide Collection database using the Basic Local Alignment Search Tool (BLAST).
